# Sunshine versus gold: The effect of population age on genetic structure of an invasive mosquito

**DOI:** 10.1002/ece3.6661

**Published:** 2020-08-19

**Authors:** Evlyn Pless, Kristen A. Hopperstad, Nicholas Ledesma, Daniel Dixon, Jennifer A. Henke, Jeffrey R. Powell

**Affiliations:** ^1^ Department of Ecology and Evolutionary Biology Environmental Science Center Yale University New Haven Connecticut USA; ^2^ Department of Entomology and Plant Pathology North Carolina State University Raleigh North Carolina USA; ^3^ California Department of Public Health Vector‐borne Disease Section Ontario California; ^4^ Anastasia Mosquito Control Saint Augustine Florida USA; ^5^ Coachella Valley Mosquito and Vector Control District Indio California USA; ^6^ Present address: USDA‐APHIS, National Veterinary Services Laboratories (NVSL) Veterinary Services Ames Iowa

**Keywords:** *Aedes aegypti*, age of population, invasive species, mosquitoes, population genetics, population structure

## Abstract

The genetic diversity and structure of invasive species are affected by the time since invasion, but it is not well understood how. We compare likely the oldest populations of *Aedes aegypti* in continental North America with some of the newest to illuminate the range of genetic diversity and structure that can be found within the invasive range of this important disease vector. *Aedes aegypti* populations in Florida have probably persisted since the 1600‐1700s, while populations in southern California derive from new invasions that occurred in the last 10 years. For this comparison, we genotyped 1,193 individuals from 28 sites at 12 highly variable microsatellites and a subset of these individuals at 23,961 single nucleotide polymorphisms (SNPs). This is the largest sample analyzed for genetic structure for either region, and it doubles the number of southern California populations previously analyzed. As predicted, the older populations (Florida) showed fewer indicators of recent founder effect and bottlenecks; in particular, these populations have dramatically higher genetic diversity and lower genetic structure. Geographic distance and driving distance were not good predictors of genetic distance in either region, especially southern California. Additionally, southern California had higher levels of genetic differentiation than any comparably sized documented region throughout the worldwide distribution of the species. Although population age and demographic history are likely driving these differences, differences in climate and transportation practices could also play a role.

## Graphical abstract

Invasive species are a threat to conservation, human health, and the economy. We present a case study showing that even within an invasive species' range, its genetic diversity and structure can vary dramatically depending on the age of the populations. Specifically, we compare ‘old’ and ‘new’ populations of the invasive mosquito *Aedes aegypti*, the primary vector of yellow fever, dengue, Zika, and chikungunya.

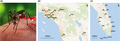

## 1 INTRODUCTION

Invasive alien species (IAS) are an ecological, economic, and health threat. They are the second most common threat associated with species that have gone extinct since the 1500s (Bellard, Cassey, & Blackburn, 2016), and their annual global cost is estimated to be more than $1.4 trillion—nearly 5% of the world economy (Pimentel et al., 2001). In the case of invasive vectors and their associated human pathogens, IAS can introduce new diseases to naïve populations and increase the range of infectious diseases (Lounibos, 2002).

The success of IAS also presents some compelling biological paradoxes and genetic complexities. Loss of genetic variation due to bottlenecks and small population size is thought to harm populations through inbreeding depression and an inability to evolve to new environments (Allendorf & Lundquist, 2003). However, some IAS not only survive bottlenecks, but they go on to flourish and outcompete the outbred and highly adapted native species. To make matters more complex, some IAS do not show lower genetic diversity at all. In fact, when an invasive population derives ancestry from multiple invasions, its genetic diversity can be even higher than any of the source populations (Allendorf & Lundquist, 2003; Hänfling, 2007). The number of founders, the number of invasions, the time since invasion, local adaptation, gene flow, and hybridization with local species are a few of the factors that can ultimately affect the genetic diversity and structure of an IAS (Allendorf & Lundquist, 2003; Hänfling, 2007).

**FIGURE 1:**
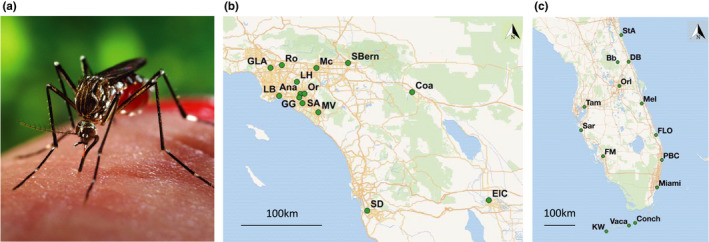
*Aedes aegypti* (a) sampling sites in southern California (b) and Florida (c), showing region size and sampling design. *Ae. aegypti* photo by James Gathany/CDC. See Table 1 for full names of each site and additional information. Maps created in QGIS; basemaps from Wikimedia Commons

We investigate the genetic diversity and structure of the invasive *Aedes aegypti* mosquito, specifically by comparing well‐established and newly founded populations in North America. *Ae. aegypti*—the primary vector of yellow fever, Zika, dengue, and chikungunya—originated in Africa and has since spread throughout much of the tropics and parts of the subtropics. *Ae. aegypti* first reached North America during the 1500s via the Atlantic slave trade, and it established overwintering populations in the US southeast that have likely persisted until today (Powell, Gloria‐Soria, & Kotsakiozi, 2018). The species is distributed in urban areas throughout the southern tier of the United States and parts of Mexico, and its average active dispersal is no greater than ~200 m (Honorio et al., 2003; Reiter, 2007; Russell, Webb, Williams, & Ritchie, 2005), but it can also disperse by “hitchhiking” via human transportation (Fonzi, Higa, Bertuso, Futami, & Minakawa, 2015; Goncalves da Silva et al., 2012; Guagliardo et al., 2014). In this study, we compare the population genetics of the likely oldest populations in continental North America (in Florida, the “Sunshine State”) with some of the youngest (in the southern portion of California, the “Golden State”; Figure 1).

With its tropical and subtropical climate, Florida has one of the highest densities of *Ae. aegypti* populations in the United States (Dickens, Sun, Jit, Cook, & Carrasco, 2018; Hahn et al., 2016) which increases risk of disease transmission, as illustrated by occasional outbreaks of *Aedes*‐borne disease (Kuehn, 2014; Likos et al., 2016; Teets et al., 2014). Populations of *Ae. aegypti* have persisted in Florida for more than 200 years, likely making them the longest established populations in continental North America; other contenders for oldest populations were eliminated by vector control in the 1950s–1960s or replaced by *Ae. albopictus* in the 1980–1990s (Lounibos, Bargielowski, Carrasquilla, & Nishimura, 2016; Slosek, 1986; Soper, 1965). Given that this mosquito has 6–12 generations per year depending on location, hundreds of years translate to thousands of generations. *Ae. aegypti* populations in southern California, on the other hand, are very young, and they face a more temperate climate which is generally predicted to be less suitable for the species (Dickens et al., 2018). *Ae. aegypti* were first reported in California in 2013 in the central California counties of Madera, Fresno, and San Mateo (Metzger, Hardstone Yoshimizu, Padgett, Hu, & Kramer, 2017). In 2014 and 2015, the species was detected in many more counties, primarily in southern California (Metzger et al., 2017).

Previous work on *Ae. aegypti* genetic diversity and structure in Florida show a variable amount of genetic differentiation across the state. Using mitochondrial DNA, Damal et al. find no differentiation between the east and west coast of Florida, whereas Hopperstad et al. report the opposite result using nine microsatellites (although with a high amount of admixture within most of the groups; Damal, Murrell, Juliano, Conn, & Loes, 2013; Hopperstad, Reiskind, Labadie, & Reiskind, 2019). Using ddRADseq, Burford Reiskind, Labadie, Bargielowski, Lounibos, and Reiskind (2018) show differentiation between the four Florida populations included in their analysis. Previous work with microsatellite and SNP chip data (Gloria‐Soria, Brown, Kramer, Yoshimizu, & Powell, 2014; Pless et al., 2017), as well as whole‐genome sequencing data (Lee et al., 2019), indicates multiple invasions of *Ae. aegypti* into California, probably at least one from the US southeast and one from the US southwest and/or northern Mexico. Southern California appears to have more genetic structure and lower genetic diversity than northern California (Lee et al., 2019; Pless et al., 2017). Few studies have explicitly examined how time since invasion affects the genetic structure of an invasive species. In one of these studies, Sherpa, Rioux, Pougnet‐Lagarde, and Després (2018) found newly established populations of *Ae. albopictus* in Europe had lower genetic diversity and higher amounts of genetic structure than *Ae. albopictus* populations established on Réunion Island in the 17th or 18th century. Additionally, geographic distance was a good predictor of genetic distance on Réunion Island, a common characteristic of populations at equilibrium known as Isolation by Distance (Wright, 1943).

Florida and southern California provide an intriguing comparison as the oldest and newest populations of *Ae. aegypti* in continental North America. We build on previous Florida work by including additional sites and incorporating both microsatellites and SNPs in the same study (microsatellites and SNPs have different strengths, and using both can provide complementary information; DeFaveri, Viitaniemi, Leder, & Merilä, 2013). We build on previous southern California work by including the most expansive study of population genetics in southern California to date, more than doubling the number of sites previously sampled. Additionally, one site, Santa Ana, was sampled in both 2015 and 2017, offering an opportunity to test whether this population is temporally stable and compare the results to the populations in Florida that were tested in multiple years (Palm Beach County and Key West).

In line with previous work (Sherpa et al., 2018) and the expectations of recent founder effects (Nei Maruyama & Chakraborty 1975), we tested the following predictions: (a) Southern California would have lower genetic diversity and a higher amount of genetic structure than Florida, (b) geographic and driving distance would be more important predictors of genetic distance in Florida than southern California, and (c) populations that were sampled more than once (in different years) are more likely to be stable in Florida than southern California.

## 2 METHODS

### 2.1 Mosquito collections

Mosquitoes from a total of 28 sites, 14 in Florida and 14 in southern California, were included in these analyses, with collection years ranging from 2006 to 2018 (Table 1, Figure 1b,c). For three populations (Palm Beach County FL, Key West FL, and Santa Ana CA), we have samples from two years, bringing our total number of “populations” analyzed to 31. Since some analyses are sensitive to large differences in sample size, populations with more than 55 individuals are represented by a random selection of 50 individuals. After this correction, the mean sample size was 38. All mosquitoes were collected as adults or eggs from traps and were shipped as adults to Yale University for analysis. No more than six individuals were used from a single ovitrap to minimize the chance of over‐sampling siblings.

**TABLE 1:** *Aedes aegypti* populations included in this study

Site	Abbreviation	Year	*N*	H_O_	H_E_	AR	*F*_IS_	New
St. Augustine, FL	StA	2017	48	0.589	0.651	4.8	0.096	Micr.
Barberville, FL*	Bb	2017	40	0.573	0.618	4.12	0.072	Both
Daytona Beach, FL*	DB	2017	44	0.650	0.607	4.27	−0.072	Both
Orlando, FL*	Orl	2014	32	0.650	0.602	4.03	−0.080	Both
Melbourne, FL*	Mel	2014	45	0.586	0.622	4.49	0.057	Micr.
Rio, FL	FLO	2014	51	0.609	0.633	4.35	0.039	Micr.
Palm Beach County, FL*	PBC13	2013	50	0.580	0.636	4.68	0.089	SNPs
Palm Beach County, FL	PBC2018	2018	50	0.624	0.640	4.78	0.025	Micr.
Miami, FL*	Miami	2011	47	0.673	0.637	4.56	−0.057	No
Vaca Key, FL	Vaca	2009	50	0.533	0.611	4.35	0.127	No
Conch Key, FL	Conch	2006	50	0.599	0.611	4.1	0.020	No
Key West, FL	KW13	2013	52	0.612	0.615	4.26	0.005	No
Key West, FL*	KW16	2016	54	0.625	0.633	4.4	0.013	No
Fort Myers, FL*	FM	2014	37	0.625	0.630	4.62	0.008	Micr.
Sarasota, FL*	Sar	2014	39	0.666	0.664	4.42	−0.002	Both
Tampa, FL*	Tam	2014	50	0.583	0.636	4.7	0.083	Both
Los Angeles, CA*	GLA	2014	6	0.556	0.540	2.75	−0.028	No
Rosemead, CA	Ro	2017	41	0.538	0.535	3.6	−0.005	Micr.
Montclair, CA	Mc	2016	30	0.479	0.504	2.67	0.050	Micr.
San Bernardino, CA	SBern	2017	48	0.481	0.517	3.57	0.070	Micr.
La Habra, CA	LH	2017	13	0.410	0.469	2.92	0.126	Micr.
Long Beach, CA	LB	2017	6	0.386	0.408	2.17	0.054	Micr.
Anaheim, CA	Ana/Ana_LC	2015	31	0.552	0.537	3.17	−0.027	No
Orange, CA	Or	2015	13	0.474	0.429	2.33	−0.106	No
Garden Grove, CA*	GG	2015	29	0.346	0.338	2.11	−0.024	No
Santa Ana, CA	SA	2015	30	0.344	0.340	2.33	−0.010	No
Santa Ana, CA	SA17	2017	33	0.524	0.520	3.46	−0.008	Micr.
Mission Viejo, CA*	MV	2015	51	0.337	0.335	2.63	−0.006	No
Coachella, CA	Coa	2017	27	0.416	0.478	3.48	0.130	Micr.
San Diego, CA*	*SD*/Cw/SY	2015	50	0.502	0.504	3.16	0.005	No
El Centro, CA	ElC	2016	46	0.515	0.536	3.81	0.040	Micr.

Note

Sampled locations, corresponding abbreviation, sampling year, number of individuals genotyped for microsatellites (*N*), observed heterozygosity, expected heterozygosity, allelic richness (*N* = 30), inbreeding coefficient, and whether the sample is being published for the first time. In the “New” column, “SNPs” means the SNP data are being published for the first time, and “Micr.” means the microsatellite data are being published for the first time. All populations were genotyped at microsatellites, and those followed by an asterisk (*) also have SNP data.

### 2.2 DNA extraction and genotyping

Whole genomic DNA was extracted from all mosquitoes using the Qiagen DNeasy Blood and Tissue kit according to manufacturer instructions, including the optional RNAse A step. As in Brown et al. (2011), all individuals were genotyped at 12 highly variable microsatellite loci: four with trinucleotide repeats (A1, B2, B3, and A9) and eight with di‐nucleotide repeats (AC2, CT2, AG2, AC4, AC1, AC5, AG1, and AG4). Any individuals that genotyped at fewer than 10 loci were excluded from analysis.

Additionally, a total of 156 individuals from ten Florida sites and four southern California sites were genotyped for single nucleotide polymorphisms (SNPs) using Axiom_aegypti, a high‐throughput genotyping chip that has 50,000 probes (Evans et al., 2015). Genotyping was conducted by the Functional Genomics Core at University of North Carolina, Chapel Hill. To prune the SNPs, we first excluded 2,166 that failed a test of Mendelian inheritance (Evans et al., 2015). Since some analyses can be confounded by SNPs in linkage disequilibrium (Alexander, Novembre, & Lange, 2009), we excluded tightly linked SNPs with Plink 1.9 using the command “‐‐indep‐pairwise 50 5 0.5” (Gloria‐Soria et al., 2018). We also excluded any SNPs that genotyped in <98% of the individuals and those with a minor allele frequency of <1%, as these could be genotyping errors, leaving 23,961 SNPs remaining for analysis.

All microsatellite and SNP data have been archived on Dryad (doi:10.5061/dryad.83bk3j9p7 and doi:10.5061/dryad.8gtht76m8). Microsatellite data for eight of the 15 southern California populations and ten of the 16 Florida populations, as well as SNP data for six of the Florida populations, are being published here for the first time (Table 1).

### 2.3 Genetic diversity

All microsatellite loci were tested for within‐population deviations from Hardy–Weinberg equilibrium and for linkage disequilibrium among loci pairs using the R package Genepop v. 1.1.4. with 10,000 dememorizations, 1,000 batches, and 10,000 iterations per batch for both tests (Raymond & Rousset, 1995). To correct for multiple testing, a Bonferroni correction was applied at the 0.05 α level of significance. Observed heterozygosity (H_O_), expected heterozygosity (H_E_), and the inbreeding coefficient (*F*_IS_) were calculated for each population using Genepop, and allelic richness was estimated by rarefaction (*N* = 30) using the software HPRARE v. 1.0 (Kalinowski, 2005). The measurements were not calculated using the SNP dataset, because the SNP chip was designed to show equal genetic diversity across different populations (Evans et al., 2015).

### 2.4 Genetic structure

We calculated pairwise genetic differentiation (*F*_ST_) for microsatellites with Genepop v. 1.1.4. and tested for significance using an exact conditional contingency‐table test with the following parameters: 10,000 dememorizations, 500 batches, and 10,000 iterations per batch (Raymond & Rousset, 1995). We calculated *F*_ST_ for SNPs using the same method, and we used 1,000 permutations to test for significance in Arlequin v. 3.5 (Excoffier, Laval, & Schneider, 2005). Within each region, we tested for a relationship between linearized *F*_ST_ (*F*_ST_/(1 − *F*_ST_)) and geographic and driving distances using a Mantel test with 9,999 permutations. Driving distance was calculated by finding the fastest driving routes between pairs of sites using Google Maps.

To explore genetic structure, we conducted twenty independent runs of STRUCTURE v. 2.3.4 for *K* = 1–12 for the complete microsatellite dataset and for each region (Pritchard, Stephens, & Donnelly, 2000). We used 600,000 generations, with the first 100,000 discarded as burn‐in. We visualized the results using the programs Clumpak and DISTRUCT v.1.1 (Kopelman, Mayzel, Jakobsson, Rosenberg, & Mayrose, 2015; Rosenberg, 2004), and we inferred the optimal value of *K* using relevant guidelines (Cullingham et al., 2020; Earl, 2012; Evanno, Regnaut, & Goudet, 2005). For the SNP dataset, we used the maximum likelihood software Admixture v. 1.3.0 and the CV error method described in the software's manual (Alexander et al., 2009). Additionally, we ran principal component analysis (PCA) for both datasets and discriminant analysis of principal components (DAPC) for the microsatellite dataset using the R package Adegenet v. 2.1.1. (Jombart, 2008). DAPC is a multivariate method for identifying genetic clusters which seeks to maximize variance between inferred groups (with inferred groups selected using a clustering algorithm, *k*‐means).

## 3 RESULTS

### 3.1 Genetic diversity

Considering each population separately, there were 372 microsatellite loci (12 microsatellites × 31 populations) we tested for deviations from the Hardy–Weinberg equilibrium and 2,112 microsatellite locus pairs we tested for linkage disequilibrium. Four out of 357 (1.1%) microsatellite loci across populations were out of Hardy–Weinberg equilibrium after a Bonferroni correction, and there were insufficient data to determine the *p*‐values for the other 15 loci. Similarly, 59 out of 1,976 (3.0%) locus pairs showed significant evidence of being in linkage disequilibrium after a Bonferroni correction, and there were not enough data to determine the *p*‐values for 136 locus pairs. These numbers are similar to what we would expect by chance, and there was no microsatellite or microsatellite pair that was consistently problematic. As such, we assumed that these are independent, neutral loci, and all 12 microsatellites were included in the analyses.

Expected heterozygosity, observed heterozygosity, and allelic richness (measures of genetic diversity) were significantly higher in Florida than in southern California (Table 2) (Student's *t* test, *p* < 10^–5^). There was no statistically significant difference in the inbreeding coefficient between the two regions on average (*p* > .05). Four Florida sites and eight southern California sites had negative *F*_IS_ values, indicating less relatedness than expected under random mating (Table 1).

**TABLE 2:** Microsatellite genetic diversity by region

Region	H_O_	H_E_	*F*_IS_	AR
Florida	0.61 ± 0.037	0.63 ± 0.017	0.026 ± 0.061	4.43 ± 0.24
S. California	0.46 ± 0.078	0.46 ± 0.077	0.017 ± 0.062	2.94 ± 0.57

Note

Observed heterozygosity (H_O_), expected heterozygosity (H_E_), inbreeding coefficient (*F*_IS_), and allelic richness (AR) estimated with rarefaction (*N* = 30 genes).

### 3.2 Genetic structure

All pairwise *F*_ST_ values (a measure of genetic differentiation) calculated with microsatellites were significantly greater than zero (*p* < .05), except for Palm Beach County 2013 and 2018 (*p* = .22) (Dryad doi:10.5061/dryad.pnvx0k6jn). The *F*_ST_ values (mean ± *SD*) among Florida sites (0.033 ± 0.018) were significantly lower than the *F*_ST_ values among southern California sites (0.23 ± 0.10) (Student's *t* test, *p* < 10^–37^). Since three of the sites are represented twice in the dataset, we repeated the analysis after excluding the older site from each pair, and the difference persisted (*p* < 10^–32^). *F*_ST_ values for pairs between Florida and southern California (0.18 ± 0.057) were significantly lower than pairs within southern California (*p* < 10^–4^). Similarly, all pairwise *F*_ST_ values calculated with SNPs were significantly greater than zero. The *F*_ST_ values (mean ± *SD*) among Florida sites (0.038 ± 0.013) were significantly lower than the *F*_ST_ values among southern California sites (0.20 ± 0.057; Student's *t* test, *p* < 10^–5^).

**FIGURE 2:**
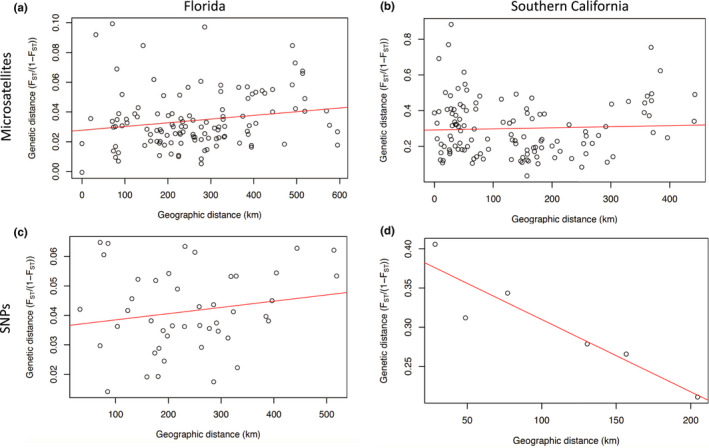
Geographic distance (km) versus genetic distance (linearized *F*_ST_ calculated with microsatellite data) for pairs of sites within (a) Florida and (b) southern California. Geographic distance versus genetic distance calculated with SNPs for pairs of sites within (c) Florida and (d) southern California. Red line shows best fit linear regression

There was a slight and marginal correlation between linearized *F*_ST_ and genetic distance (Mantel's *R* = 0.18, *p* = .052; Figure 2a) and driving distance within Florida (Mantel's *R* = 0.19, *p* = .062) using microsatellites. According to a Mantel test, there was a negative relationship between linearized *F*_ST_ and geographic distance within southern California (Mantel's *r* = −0.41, *p* = .009), and no significant relationship between linearized *F*_ST_ and driving distance (Mantel's *R* = −0.37, *p* = .98). However, a plot of geographic distance versus genetic distance suggests a “U” shape rather than a negative relationship (Figure 2b). Using SNPs, there was no significant correlation between geographic and genetic distance within Florida (Mantel *R* = 0.18, *p* = .21; Figure 2c) or within southern California (Mantel *R* = −0.93, *p* = .96), although the relationship appears negative in a plot of geographic versus genetic distance (Figure 2D).

**FIGURE 3:**
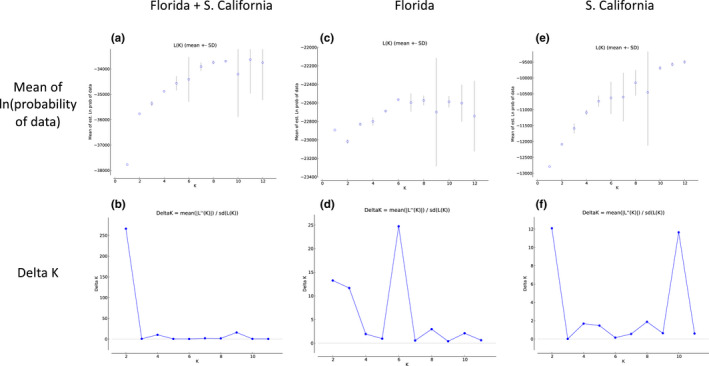
Mean of estimated ln(probability) of data versus *K* for (a) full dataset, (c) Florida, and (e) southern California. Delta *K* versus *K* for (b) full dataset, (d) Florida, and (f) southern California

**FIGURE 4:**
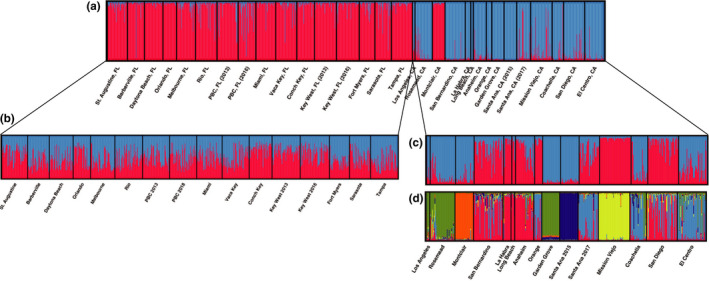
STRUCTURE plots for microsatellite data, where each vertical bar represents an individual, and the colors of the bar represent what proportion of each individual's ancestry is attributable to each of the *K* theoretical genetic clusters. (a) Full dataset (*K* = 2, most likely), (b) Florida *K* = 2 showing the high amount of admixture in Florida populations (*K* = 1 is most likely), (c) southern California (*K* = 2, most likely), and (d) southern California (*K* = 5). While this method cannot distinguish any of the Florida populations, it can differentiate between many of the southern California populations

**FIGURE 5:**
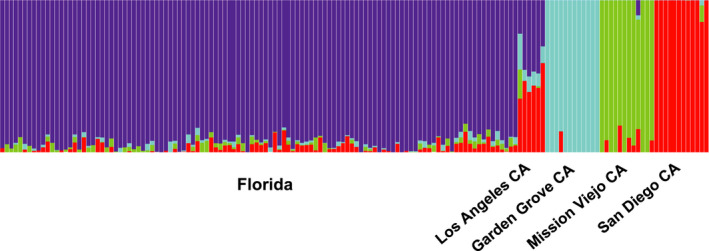
Admixture plot using SNP dataset. The fraction of each vertical bar assigned to each color represents the proportion of that individual's ancestry attributable to each of the *K* theoretical genetic clusters. *K* = 4 was selected as most likely by the CV error method provided in program's literature

Bayesian clustering analysis of microsatellite data found two primary groups in the full dataset (Figure 3a,b), which correspond to Florida and southern California with the exception of Montclair CA (Figure 4a). However, at higher *K* levels, Montclair clusters with Mission Viejo CA (e.g. *K* = 3) or as its own group (e.g. *K* = 5) (not shown). Within Florida, Bayesian clustering analysis identified one group (Figure 3c,d); we show *K* = 2 to illustrate the high level of admixture across all populations (Figure 4b). The Evanno et al. method does not consider *K* = 1, so it identified 6 groups in the Florida data (2005; Figure 3d). We ruled out this possibility because *K* = 1 has a higher probability than *K* = 2 (Figure 3c), and the Delta *K* peak at *K* = 6 is a small value, 25 (Figure 3d; Cullingham et al., 2020). Within southern California, Bayesian clustering analysis found two primary groups (Figure 3e,f), which roughly separate Los Angeles, Rosemead, Montclair, Garden Grove, Santa Ana 2015, Coachella, and El Centro from the other eight populations (Figure 4c). However, higher K values show remarkably fine‐scale population structure among many of the populations (e.g. *K* = 6 in Figure 4d). Four genetic groups were detected in the SNP dataset by Admixture's CV Error method for selecting the best *K*. The four populations cleanly delineate Florida, Mission Viejo, Garden Grove, and San Diego, while Los Angeles appears to be an admixed group, sharing ancestry primarily with Florida and San Diego (Figure 5).

**FIGURE 6:**
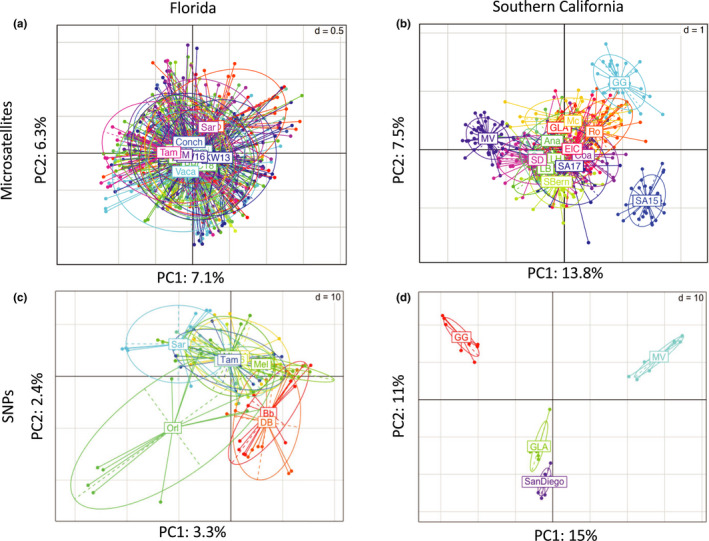
Principal component analysis of microsatellite data for (a) Florida and (b) southern California and of SNP data for (c) Florida and (d) southern California. Ellipses indicate the distribution of individuals within each group. While the Florida populations are overlapping in PC spaces (indicating little genetic structure), the southern California populations are more differentiated. See Table 1 for full names of each site and additional information

**FIGURE 7:**
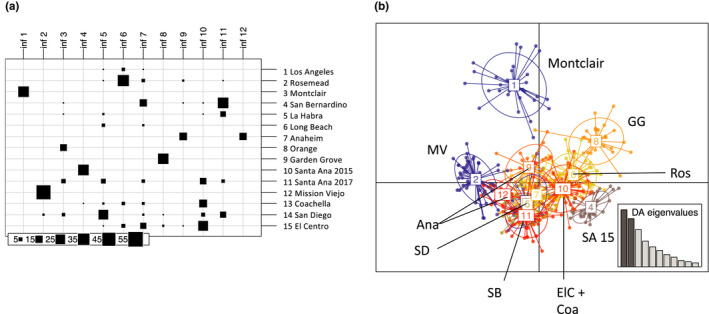
Discriminant analysis of principal components for southern California microsatellite data. (a) *k*‐means clustering of southern California individuals into 12 inferred groups. (b) DAPC plot for the 12 inferred groups

For Florida, PCA showed essentially no differentiation among populations using microsatellites and only a small amount of differentiation among groups using SNPs (Figure 6a,c). Although DAPC found some spread among inferred groups using microsatellites, the *k*‐means clustering algorithm split the individuals into inferred groups in a way that did not align with their original sampling locations (not shown). For southern California microsatellites, PCA showed differentiation among some populations, especially Santa Ana 2015, Garden Grove, and Mission Viejo (Figure 6b). DAPC produced a similar result, except with more differentiation among populations, and it showed Montclair as the primary outlier (Figure 7). The populations in the top‐right portion of the DAPC plot generally correspond to one of the inferred population groups in STRUCTURE at *K* = 2 (the blue group in Figure 4b), and the bottom‐left portion corresponds to the other group (the red group in Figure 4b). Using SNP data, PCA cleanly separates the four southern California populations; Los Angeles and San Diego are close in PC space, while Garden Grove and Mission Viejo are set apart (Figure 6d).

In terms of temporal stability, there was no indication of genetic difference between the two years for Palm Beach County or Key West using STRUCTURE, PCA, or DAPC. The pairwise *F*_ST_ between Palm Beach County 2013 and 2018 was not significantly different than zero, and the pairwise *F*_ST_ between Key West 2013 and 2016 was 0.018 (lower than the intra‐Florida mean of 0.033). However, in southern California, Santa Ana 2015 and Santa Ana 2017 cluster separately using STRUCTURE (Figure 4c,d) and *k*‐means clustering (Figure 7a), and their pairwise *F*_ST_ is 0.28 (even higher than the intra‐southern California mean of 0.23). Additionally, the mean pairwise *F*_ST_ values within southern California sites collected in 2014 and 2015 is 0.30 ± 0.12, which is significantly higher than the mean pairwise *F*_ST_ values within sites collected in 2015 and 2016 (0.18 ± 0.065) (Student's *t* test, *p* = .0003).

## 4 DISCUSSION

Invasive alien species (IAS) create environmental, economic, and health problems around the globe, and the *Aedes aegypti* mosquito provides a clear example of this phenomenon. This species evolved from a forest‐dwelling mosquito in Africa to an anthrophilic disease vector found on every continent but Antarctica. It is the primary vector for numerous debilitating and costly diseases; the global burden dengue alone is immense with around 100 million new cases each year (Bhatt et al., 2013). Several characteristics affect the success of an invasion as well as the genetic structure and diversity of the invaded species. We examine the most well‐established (>200ya) and the most recently established (<10ya) populations of *Ae. aegypti* in continental North America and find a remarkable difference in genetic diversity and population structure. We predicted that the new populations in southern California would have more signs of recent founder effect and bottlenecks than the old populations from Florida. Specifically, we expected new populations to have lower genetic diversity, higher genetic differentiation among populations, less temporal stability, and less evidence of Isolation by Distance. Although these expectations were generally met, there were also surprises, including the dramatic and unique extent of differentiation and structure in southern California.

In line with our expectations, the newly established populations in southern California had significantly lower genetic diversity (allelic richness, observed heterozygosity, and expected heterozygosity), and more southern California populations showed evidence of inbreeding (eight in southern California vs. four in Florida; Table 1). Southern California had more genetic structure and higher pairwise *F*_ST_ (Figures 4 and 6), and there was no increase in genetic distance with geographic (or driving) distance (Figure 2). While the two samples in Florida that were resampled in separate years appeared to be temporally stable, the population resampled in southern California showed high genetic differentiation and change over just two years (e.g. Figure 4c,d).

Contrary to expectations, Florida had no relationship between driving distance and genetic distance and only a marginal increase in genetic distance with geographic distance (Figure 2). Even more surprising, the high pairwise *F*_ST_ values and genetic structure within southern California are unlike any other region examined on a global scale using the same genetic markers (Gloria‐Soria et al., 2016). For example, the mean pairwise *F*_ST_ value within southern California (0.23) is higher than mean *F*_ST_ values between Africa and other continents (0.11–0.14).

Overall the results allude to very different invasion timelines and histories for these two regions. Southern California shows signs of recent bottleneck, inbreeding, serial founder effect, and possibly multiple invasions from different regions. Florida is also in the invasive range of *Ae. aegypti*, and indeed its allelic richness is lower than populations sampled in Africa (Gloria‐Soria et al., 2016). Although Florida populations were subject to bottlenecks, ~2,000–5,000 generations (assuming ten generations/year) of mutation and admixture—likely involving numerous introduction events from Africa—have muted those effects, especially compared to southern California. Additionally, a relatively high amount of gene flow (≥1 individual per generation) likely still occurs among most Florida populations preventing distinct population structure from forming (Nathan, Kanno, & Vokoun, 2017). This gene flow is probably mediated by stochastic human movement, since geographic and driving distance are not good predictors of genetic distance.

In addition to time since invasion, the number of invasions, number of propagules during invasions, and other population bottlenecks or demographic history events could affect the genetic diversity and structure patterns we see here. Although we did not detect differences in effective population size or number of recent bottleneck events between these two regions (results not shown), more work and demographic history inference is needed, and we are currently analyzing these regions further in the context of North America more broadly.

**FIGURE 8:**
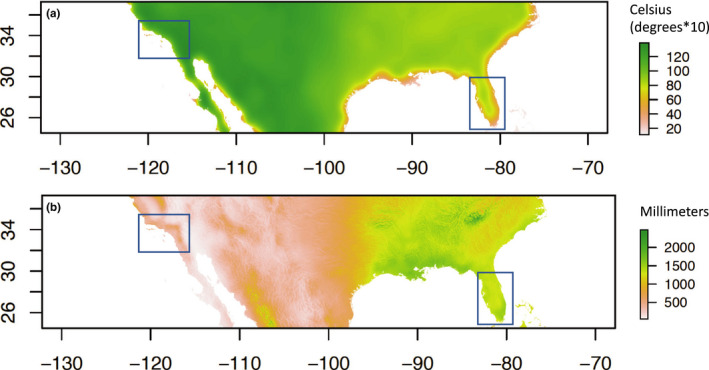
Differences in climate between the two sample regions (boxes) as shown by two examples: (a) daily temperature range and (b) mean annual precipitation. Green shows high temperature range/precipitation, and red shows low temperature range/precipitation. Derived from CHELSA climate data (Karger *et al*. 2017)

Although we believe time since invasion is the most important factor driving the differences between Florida and southern California, the differences in climate between the regions could also have an effect. Florida is more tropical: It has more precipitation, higher humidity, a smaller daily temperature range, warmer winters, and wetter summers (Figure 8). It is rated as higher habitat suitability than southern California in all studies we are aware of (Dickens et al., 2018), and there is evidence genetic differentiation can change depending on season (Huber et al., 2002; Sayson, Gloria‐Soria, Powell, & Edillo, 2015). The more tropical climate of Florida may promote year‐round persistence and breeding of *Ae. aegypti*. Indeed the temporal instability of Santa Ana CA may indicate the population decreased to low levels in the winter, and perhaps even went locally extinct, before getting reseeded the following year. Differences in transportation networks, perhaps combined with differences in climate and seasonal events, could also affect the genetic connectivity among the populations.

This work builds on previous studies from Florida and southern California by increasing the number of individuals and sites sampled, as well as the number of genetic markers. Our work supports previous findings of minimal structure (Damal et al., 2013) and a high degree of admixture in Florida (Hopperstad et al., 2019). Unlike a previous study, we find almost no evidence of Isolation by Distance in Florida (Hopperstad et al., 2019). Additionally, a geographically limited study found significant differentiation among four Florida populations (Apopka, Kissimmee, Fort Myers, and Key West) using ddRADseq (Burford Reiskind et al., 2018). The individuals included in these analyses were generation F2 or F3 from laboratory colonies and were sampled differently than our samples, as the purpose of the study was to find genomic differences in mating behavior (not characterize genetic structure). In terms of southern California, this paper greatly expands the number of populations analyzed in the region, and it bolsters the finding of high levels of genetic structuring in southern California (Lee et al., 2019; Pless et al., 2017). Further, we provide new evidence of local temporal change in southern California in the first few years after the first detection in the area. Specifically, Santa Ana shows genetic change after just two years, and there is a decrease in pairwise *F*_ST_ among the 2016–2017 sites, perhaps indicating a diminishing of the most extreme effects of bottleneck.

Another study compared genetic structure and diversity of new (Europe) and old (Réunion Island) populations of a similar species, *Ae. albopictus* (Sherpa et al., 2018). Like these authors, we found higher diversity and lower amounts of structure in the older invasive populations; however, we did not find strong evidence of Isolation by Distance in the older populations (Sherpa et al., 2018). This could be caused by differences in the organism (*Ae. aegypti* is more anthrophilic and has a shorter active dispersal range than *Ae. albopictus*) or the regions (Florida is larger and has different transportation patterns than Réunion Island) (Chouin‐Carneiro et al., 2016; Vavassori, Saddler, & Müller, 2019).

We provide this case study to illustrate that even within its invasive range, the population genetics and structure of an IAS can vary dramatically. As such, a “one size fits all” control measure may not be appropriate for controlling an invasive species; rather the control methods should be tailored to the region in question and may need to be adjusted over time. Moreover, the marked differences between the two regions considered here evoke the diversities of invasion histories and novel environment *Ae. aegypti* has experienced during its global expansion. The unusual genetic patterns in southern California compared to other regions around the world make it especially intriguing for further study.

## CONFLICT OF INTEREST

The authors have no competing interests to declare. However, we would like to include this disclaimer due to an author affiliation: The findings and conclusions in this publication are those of the authors and should not be construed to represent any official USDA or US Government determination or policy.

## AUTHOR CONTRIBUTION

**Evlyn Pless:** Conceptualization (equal); Data curation (lead); Formal analysis (lead); Investigation (lead); Methodology (lead); Project administration (supporting); Visualization (lead); Writing‐original draft (lead). **Kristen A. Hopperstad:** Resources (equal); Writing‐review & editing (equal). **Nicholas Ledesma:** Resources (equal); Writing‐review & editing (equal). **Daniel Dixon:** Resources (equal); Writing‐review & editing (equal). **Jennifer A. Henke:** Resources (equal); Writing‐review & editing (equal). **Jeffrey Powell:** Conceptualization (equal); Funding acquisition (lead); Project administration (lead); Supervision (lead).

### OPEN RESEARCH BADGES

This article has earned an Open Data Badge for making publicly available the digitally‐shareable data necessary to reproduce the reported results. The data is available at Microsatellite dataset: https://doi.org/10.5061/dryad.83bk3j9p7, SNP dataset (unfiltered and filtered): https://doi.org/10.5061/dryad.8gtht76m8.

## Data Availability

Microsatellite data: Dryad https://doi.org/10.5061/dryad.83bk3j9p7; SNP data (filtered and unfiltered): https://doi.org/10.5061/dryad.8gtht76m8
